# The Serum Analysis of Dampness Syndrome in Patients with Coronary Heart Disease and Chronic Renal Failure Based on the Theory of “Same Syndromes in Different Diseases”

**DOI:** 10.1155/2017/3805806

**Published:** 2017-06-21

**Authors:** Yiming Hao, Xue Yuan, Peng Qian, Guanfeng Bai, Yiqin Wang

**Affiliations:** ^1^Shanghai Key Laboratory of Health Identification and Assessment and Laboratory of TCM Four Diagnostic Information, Shanghai University of Traditional Chinese Medicine, Shanghai 201203, China; ^2^Shanghai Haohai Biological Technology Co., Ltd., Shanghai 200052, China; ^3^Xinmi Hospital of Traditional Chinese Medicine, Xinmi, Henan 452370, China

## Abstract

**Aim:**

To analyze the serum metabolites in patients with coronary heart disease (CHD) showing dampness syndrome and patients with chronic renal failure (CRF) showing dampness syndrome and to seek the substance that serves as the underlying basis of dampness syndrome in “same syndromes in different diseases.”* Methods*. Metabolic spectrum by GC-MS was performed using serum samples from 29 patients with CHD showing dampness syndrome and 32 patients with CRF showing dampness syndrome. The principal component analysis and statistical analysis of partial least squares were performed to detect the metabolites with different levels of expression in patients with CHD and CRF. Furthermore, by comparing the VIP value and data mining in METLIN and HMDB, we identified the common metabolites in both patient groups.

**Results:**

(1) Ten differential metabolites were found in patients with CHD showing dampness syndrome when compared to healthy subjects. Meanwhile, nine differential metabolites were found in patients with CRF showing dampness syndrome when compared to healthy subjects. (2) There were 9 differential metabolites identified when the serum metabolites of the CHD patients with dampness syndrome were compared to those of CRF patients with dampness syndrome. There were 4 common metabolites found in the serums of both patient groups.

## 1. Introduction

“Syndromes” are a typical concept in Traditional Chinese Medicine (TCM). It is the core of the disease development discipline in TCM. The complexity and integrity of syndromes determine the fact that a certain syndrome is not the result of the quantitative accumulation of individual change, but the outcome of comprehensive functions from multifactors. Thanks to the development in science and technology, extensive and thorough study about “syndromes” has been able to conduct recently; for example, the nature of syndromes has been investigated by applying multiomics approach using system biology theory.

Metabolomics are a high throughput omics platform to obtain large amount of data of metabolism. All the data processed by bioinformatics technique and various statistic methods can be used to seek underlying mechanism that leads to the variation between biological metabolites [1, 2]. Metabolomics study utilizes a bottom-up approach. By diversified comprehensive analysis, final metabolites can provide a complete set of information to the change of the whole organisms. It is in accordance with the concept of wholism and selecting treatment according to syndrome in TCM and avoids the disadvantage of using single or few indexes to study certain pathological and physiological change [3]. Currently, the major analysis methods in metabolomics include NMR, GC-MS, and LC-MS. NMR and GC-MS are more extensively utilized than LC-MS. Although the sample treatment for NMR test is less complicated, the detection sensitivity of NMR is much lower and the cost is relatively higher. Overall, the sensitivity of GC-MS is the highest; therefore, its application has been increased gradually.

The life style change due to the development of economy, along with the population aging, has great altered CHD epidemics. The morbidity and mortality of CHD have increased year by year, and the trend is found among younger generation. When looking through the CHD-related syndromes literature in TCM collected over the past 40 years, we found that patients with phlegm-dampness syndrome were 13.48% among 34640 patients with CHD, and it was just a little bit less than 15.02%, which is the highest ratio of patients with stagnant blockade of heart blood [4].

Research has shown that the incidence of CRF has increased noticeably recently. The prevention and control of CRF have become a big challenge. The modern Chinese medicine indicated that the initiation and progression of CRF were closely related to the pathogenic dampness because CRF appeared as the pattern of syndromes with excess in superficiality, such as damp-heat, damp-turbidity, and damp-stasis [5].

Hence, we hypothesize that dampness syndrome is the common syndrome in CHD and CRF and we would like to pursue the nature of dampness syndromes in patients with CHD and CRF since very few data is available. In this study, the serum samples of patients with CHD or CRF showing dampness syndrome were collected. The metabolites and metabolic mechanism of dampness syndrome were investigated by GC-MS omics. The discovery of our study will provide the preliminary research data for the material basis study of dampness syndrome.

## 2. Clinical Material

Fifty-eight patients with CHD were from Affiliated Hospital of Shanghai University of Traditional Chinese Medicine. Sixty-two patients with CRF were from Longhua Hospital and Shuguang Hospital affiliated to Shanghai University of TCM. All patients were admitted to hospital from September 2011 to July 2012. Serums of patients were collected. The forms of heart informatics characteristics of interrogation and clinical questionnaire of CRF were filled. Patients were divided into two groups according to the common differentiation method of TCM, dampness syndrome group and nondampness syndrome group, by two skillful TCM doctors. For CHD, there are 29 patients in dampness syndrome group: 16 male patients and 13 female patients with the average age of 72.3 ± 12.67. In the nondampness syndrome group, there were 29 patients: 18 male patients and 11 female patients with the average age of 69.41 ± 12.45. For CRF, 32 patients were in the dampness syndrome group, 20 male patients and 12 female patients with the average age of 53.37 ± 17.18, while there were 30 patients in the nondampness syndrome group, 14 male patients and 16 female patients with the average age of 52.16 ± 14.21. Twenty-five healthy staff in Shanghai University of Traditional Chinese Medicine without any organic lesion in systemic were selected as normal controls and their serum samples were collected.

## 3. Methods

### 3.1. The Standard of Diagnosis, Admission, Elimination, and Rejection

The diagnostic criteria of CHD are referred to the joint report on standardized clinical nomenclature of International Society of Cardiology and Society and World Health Organization, “the nomenclature and diagnostic criteria of ischemic heart disease” [6]. The diagnostic criteria of CRF are referred to the standard set by the professional group of kidney disease in editorial board of Chinese Journal of Internal Medicine in 1993 [7] and K/DOQI (kidney diseases and Dialysis quality of life guidance) set by American Kidney Fund in 2002 [8]. The TCM syndrome differentiation criteria of CHD are set according to the related chapters covering differentiated syndromes about CHD of TCM in the books of* The Syndrome Criteria of Pectoralgia, Chest Tightness, Palpitation, Shortness of Breath and Weakness of Coronary Heart Diseases Differentiated by TCM* [9] and* Chinese Traditional Medicine New Drug Clinical Research Guiding Principle* [10] edited by Cardiovascular Society of Chinese Integrative Medicine in 1990. The TCM syndrome differentiation criteria of CRF are set according to the related chapters covering differentiated syndromes about CRF of TCM in the book of* Chinese Traditional Medicine New Drug Clinical Research Guiding Principle* edited by Cardiovascular Society of Chinese Integrative Medicine [10].

The admission criteria for the patients with CHD are based on the diagnostic criteria of Western medicine and differentiation syndromes in TCM for CHD. The admission criteria for the CRF are based on the diagnostic criteria of Western medicine and differentiation syndromes in TCM for CRF. The patients with CRF caused by acute or chronic glomerular nephritis were mainly selected. The patient age range was from 25 to 75.

The elimination criteria of CHD were described as follows: (1) patients associated with heart failure; (2) patients with lung, kidney, blood, endocrine, metabolism, and gastrointestine primary diseases or mental illness; (3) patients during pregnancy or breastfeeding; and (4) patients in an allergic constitution. The elimination criteria of CRF were described as follows: (1) patients with heart, liver, lung, endocrine, blood, metabolism primary diseases, severe gastrointestine primary diseases, or mental illness; (2) patients with CRF caused by the extrarenal diseases; (3) patients with CRF and who require dialysis therapy; (4) patients during pregnancy or breastfeeding; and (5) patients in allergic constitution or with drug allergy.

The criteria of rejection included (1) patients without complete clinical data, either due to incomplete collection or data missing and (2) patients with discrepancy results on differentiated syndromes in TCM when diagnosed by two TCM doctors.

### 3.2. Main Reagents and Instruments

#### 3.2.1. Main Reagent

N-caproic acid, N,N-dimethylformamide, N,O-bis(trimethylsilyl)trifluoroacetamide, anhydrous ethanol, pyridine, ethyl chloroformate (ECF), chloroform (CHCl3), sodium hydroxide, anhydrous sodium sulfate, methoxamine hydrochloride (97%), and trypan blue stain.

#### 3.2.2. Main Instrument and Equipment

  Freeze dryer: Free Zone 12 L vertical freeze dryer, USA, Labconco
  Ultrasonic cracker: SONICS 750, USA, SONICS
  Vortex mixer: XW-80A, China, Shanghai Medical University Instrument Factory
  High speed refrigerated centrifuge: Avanti J-30, USA, Beckman Kurt, Hongkong
  Gas chromatography-quadrupole mass spectrometer: Thermo Trace DSQ, USA, Thermo
  Column: TR-5ms, 60 m *∗* 0.25 mm *∗* 0.25 m, USA, Thermo

### 3.3. Experimental Methods

The sample collecting method was referred to the literature [11]. Briefly, blood was drawn from the peripheral vein when patients were in fasting condition. The blood was kept at 4°C for more than 2 hours, before centrifugation at 3000 rpm/min to obtain the serum, 4°C.

For sample derivation, proteins were first removed by lyophilization and then processed with silane derivative method [12].

#### 3.3.1. GC-MS Detection

*The Chromatographic Condition*. Specifications of chromatographic column were 60 m × 0.25 mm i.d. × 0.25 *μ*m, TR-5MS. The initial temperature was set to 80°C and kept for 2 minutes before ramped up to 140°C at a rate of 10°C/min. Then the temperature continued to rump up to 240°C at a rate of 4°C/min and to 280°C at a rate of 10°C/min. Hold at 280°C for 10 minutes. The carrier gas was He with a flow rate of 1.0 mL/min. The temperature of the injection port was 280°C and the injection volume was 0.2 *μ*L.

*The Condition of Mass Spectrometry (MS)*. The solvent delay was 8 minutes. The temperature of ion source was 250°C and the temperature of ports was 280°C. MS scanning range was from 40 to 500 Da by *M*/*Z*. The ionization mode was EI. The electron energy was 70 eV.

Each sample was injected three times continuously. Every serum sample processed by silane derivative method was divided into 3 identical aliquots for injection in order to to avoid accidental error.

### 3.4. Data Processing, Statistical Analysis and Substance Identification

Capric acid was used as internal control for normalization. Serum samples and tongue coating without detected internal control were removed. Manual and automatic integration were combined and applied to complete auxiliary integral followed by noise filtering, peak alignment, peak detection, and normalization sequentially.

The principal component analysis and statistical analysis of partial least squares were performed and the possible substances were mined out by searching the METLIN database on their mass charge ratio. Metabolites were identified by comparing NIST MS database according to the retention time of differentiated substances. The related metabolic pathways of identified substances were examined by inquiring Kyoto genome and genome database (KEGG).

## 4. Results

### 4.1. Differential GC-MS Spectrum

**Figure 1:**
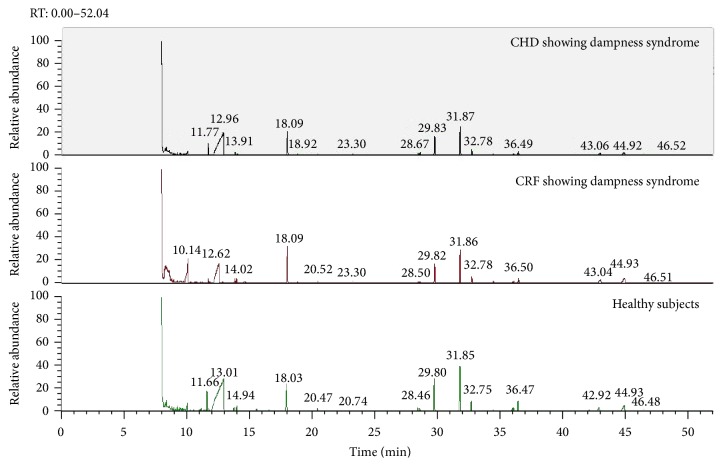
GC-MS diagram of serums from patients with CHD and CRF showing dampness syndrome and healthy subjects.

Serums from healthy subjects and patients with CHD and CRF showing dampness syndrome were examined by GC-MS. One of the differential GC-MS spectra was shown in Figure 1.

### 4.2. The Discrimination Analysis of Partial Least Squares (PLS-DA)

**Figure 2:**
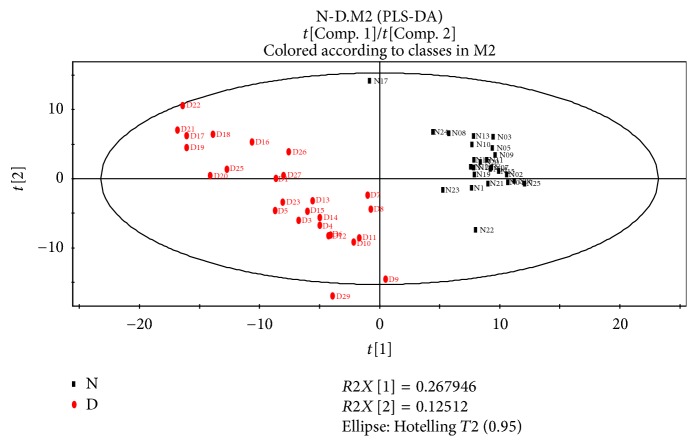
PLS-DA diagram of serums from patients with CHD showing dampness syndrome and healthy subjects (N: healthy subjects, D: patients with CHD showing dampness).

**Figure 3:**
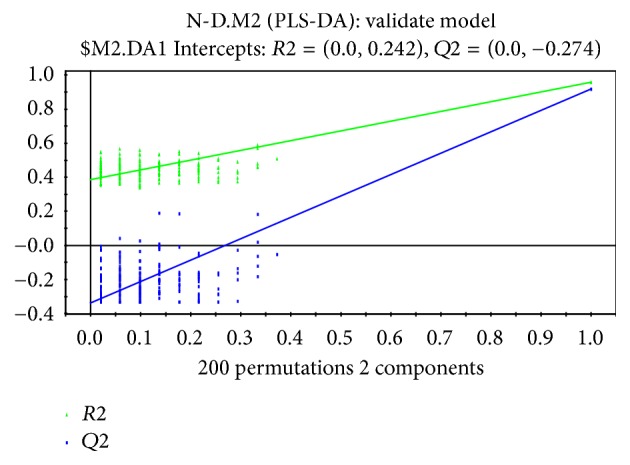
Cross validation diagram of serums from patients with CHD showing dampness syndrome and healthy subjects (cumulative interpretation rate: *R*2*X* = 0.393, supervision model explanation rate: *R*2*Y* = 0.931, and model prediction rate: *Q*2 = 0.894).

**Figure 4:**
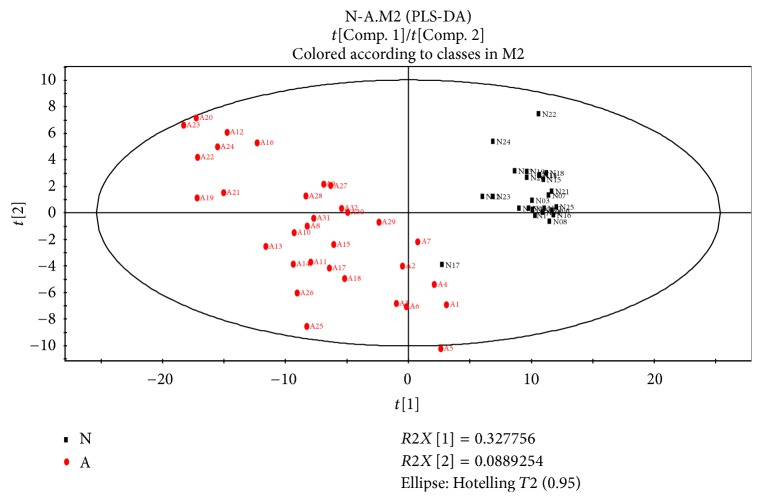
PLS-DA diagram of serums from patients with CRF showing dampness syndrome and healthy subjects (N: healthy subjects, A: patients with CRF showing dampness).

**Figure 5:**
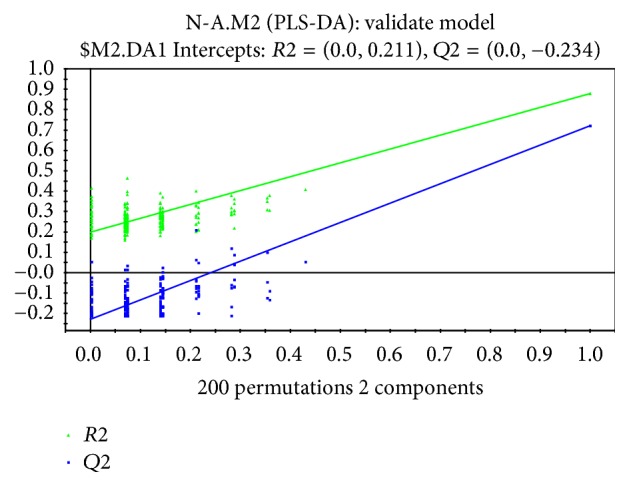
Cross validation diagram of serums from patients with CRF showing dampness syndrome and healthy subjects (cumulative interpretation rate: *R*2*X* = 0.417, supervision model explanation rate: *R*2*Y* = 0.879, and model prediction rate: *Q*2 = 0.720).

**Figure 6:**
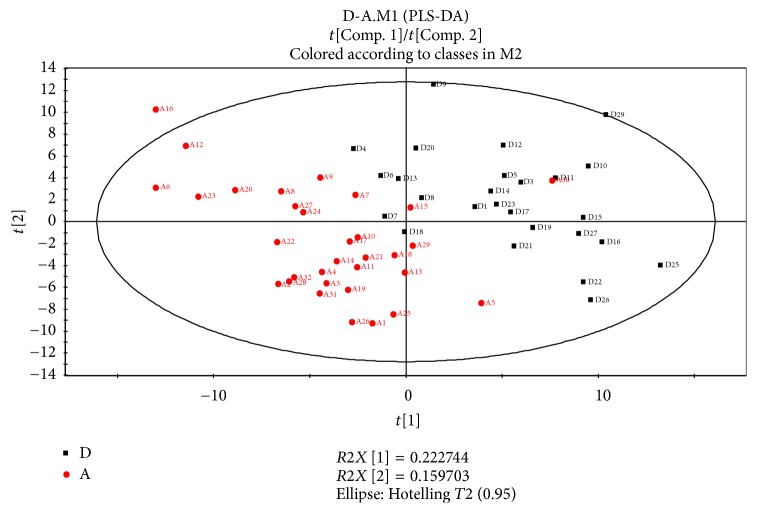
PLS-DA diagram of serums from patients with CHD and CRF showing dampness syndrome (D: patients with CHD showing dampness, A: patients with CRF showing dampness).

**Figure 7:**
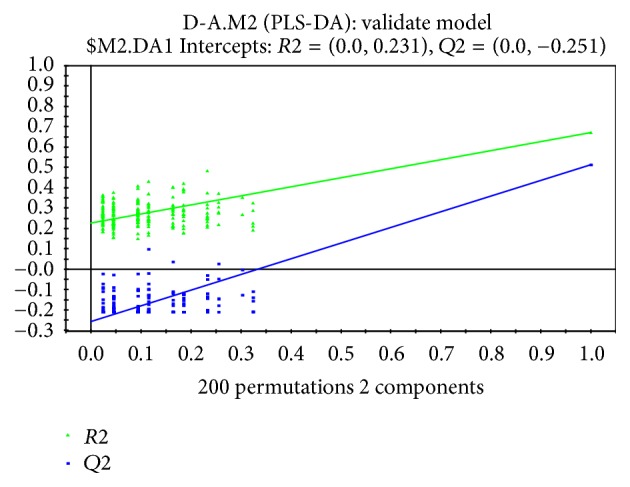
Cross validation diagram of serums from patients with CHD and CRF showing dampness syndrome (cumulative interpretation rate: *R*2*X* = 0.382, supervision model explanation rate: *R*2*Y* = 0.671, and model prediction rate: *Q*2 = 0.514).

To discriminate the difference among groups, the statistical method of PLS-DA was applied to do modeling analysis with the first and second principle components of samples and the effectiveness of models was examined by alignment experiments. There are differences in serum components between patients with CHD and CRF showing dampness syndrome and healthy subjects, which were obvious and could be distinguished easily. Furthermore, the score of cross validation diagram was relatively high (Figures 2, 3, 4, and 5). One point overlapped between serum samples from patients with CHD and CRF showing dampness syndrome; however, the difference among these two group patients was significant and could be easily discriminated. The score of cross validation diagram was also relatively high (Figures 6 and 7).

### 4.3. The Screening of the Differential Peaks and Their Corresponding Metabolites

The statistical examination of two-sample Welch *t*-statistics was applied for the peak value of two groups. The differential peaks with *P* < 0.05 and VIP ≥ 1 were selected. There were 10 differential peaks existing in the serums of patients with CHD showing dampness syndrome compared to the serums of healthy subjects (Table 1). There were 9 differential peaks existing in the serums of patients with CRF showing dampness syndrome compared to the serums of healthy subjects (Table 2). Finally, there were 9 differential peaks existing in the serums of patients with CHD showing dampness syndrome compared to those from patients with CRF showing dampness syndrome (Table 3).

The corresponding metabolites based on the differential peaks were identified through searching the METLIN and HMDB database (Tables 4, 5, and 6).

The metabolic pathways were identified and presented in Table 7, in which parts of differential metabolites from serums of patients with CHD and CRF showing dampness syndrome were involved.

### 4.4. The Screening of the Same Peaks and Their Corresponding Metabolites

The statistical examination of two-sample Welch *t*-statistics was applied for the peak values of group with dampness syndrome of CHD and group of CRF. The same metabolites were sorted out through aligning from the minimum value based on VIP value. Four peaks from two groups' serums were selected and substances were identified through searching the METLIN and HMDB database (Table 8). These substances could be the common material bases for the dampness syndrome of CHD and dampness syndrome of CRF.

The metabolic pathways were identified and presented in Table 9, in which parts of the same metabolites from serums of CHD and CRF patients with dampness syndrome were included.

## 5. Discussion

Currently, there are few studies reporting about dampness syndrome. From the view of disease category, there are studies on dampness syndrome towards hypertension, chronic hepatitis B, diabetes, and other metabolic diseases; and biochemical indexes are the major investigation objectives. Our group has been investigated dampness syndrome of CHD, CRF, and chronic gastritis for a long time. We studied dampness syndrome through various approaches in epidemiology and objective information about 4 diagnostic methods of TCM, proteomics, and metabolomics. We have screened and identified differential proteins in patients with CHD and CRF showing dampness syndrome by proteomics technique [13, 14], which offered new insights about dampness syndrome of TCM. Meanwhile, it is beneficial to apply metabolomics technique on objectification of dampness syndrome and the comparative research on dampness syndrome of various diseases.

The results from current study showed that there were 10 differential metabolites existing in serums of CHD patients with dampness syndrome compared to healthy subjects. These metabolites were 2-oxo-3-methylvaleric acid, 3-hydroxy-DL-kynurenic acid, vinylacetylglycine, pregnenolone, nitrotyrosine, 1-methyl adenosine, oxoadipic acid, glyceric acid, citric acid, and isobutyric acid.

2-oxo-3-Methylvaleric acid is the metabolic product of isoleucine. Isoleucine is one of the essential amino acids in the body. It can work with leucine and valine to restore the muscle, control blood glucose, and help burning visceral fat to provide energy for body tissues. 3-Hydroxy-DL-kynurenic acid is the metabolic product of tryptophan, which is an essential amino acid. It is mainly involved in the regeneration of plasma proteins in human body. Oxoadipic acid is the degraded product of lysine. Glyceric acid is involved in the metabolic pathway of glycine and serine. Vinylacetylglycine is an acyl amino acid. Fatty acid is a metabolic product. The different levels of these amino acids found in CHD patients proved that there are lipid metabolism and energy metabolism abnormality in patients with dampness syndrome. Moreover, citrate acid is the important component of tricarboxylic acid cycle (TCA) in energy metabolism, and the abnormal level of citrate acid in patients of CHD indicated that the balance in TCA has been broken. Meanwhile, the endogenous sex hormone level in patients with CHD was abnormal. It has been reported that the level of estradiol declined and testosterone elevated in postmenopausal female patients with CHD [15].

There were 9 differential metabolites existing in the serums of patients with CRF showing dampness syndrome comparing with the serums of healthy subjects. They were 2-oxo-3-methylvaleric acid, dihydroxyacetone phosphate, adenosine, vinylacetylglycine, imidazoleacetic acid, pyridoxal, 5-hydroxymethyluracil, L-tyrosine, and orotic acid.

Dihydroxyacetone phosphate is an important intermediate product in the biosynthesis of lipid and glycolysis. It is involved in degradation of fructose and mannose, biosynthesis of phospholipids, metabolism of glyceric acid, glycolysis, and gluconeogenesis. Patients with CRF often have disorder of glucose metabolism, characterized by diabetes-like curve of glucose tolerance and insufficient gluconeogenesis. Moreover, hypoglycemia might occur in patients in the stage of uremia. Adenosine and adenosine derivatives play an important role in the construction of DNA and RNA in the energy transferring process when adenosine triphosphate (ATP) is converted to adenosine diphosphate (ADP) and in cyclic adenosine monophosphate (cAMP) signal transduction. Pyridoxal can be converted to pyridoxal phosphate, which can work as coenzyme to participate in the synthesis of amino acids, neurotransmitters (serotonin, norepinephrine), sphingolipids, 5-aminolevulinic acid, and so forth. Imidazoleacetic acid is the metabolic product of histidine.* L*-Tyrosine participates in the biosynthesis of catecholamine other than its own metabolism.

In summary, various disorders of amino acid and lipid metabolism do exist in patients with CHD showing dampness syndrome. Glucose and energy metabolisms are often found abnormal in these patients. The main disorders including glucose, lipid and amino acids metabolisms, and biosynthetic abnormality of catecholamine are found in patients with CRF showing dampness syndrome.

There were 9 differential metabolites found in serums of patients with CHD showing dampness syndrome compared to patients with CRF showing dampness syndrome. They were retinoic acid, tyramine, 5,8-tetradecadienoic acid, citrate acid, 8-hydroxyguanine, arachidonic acid, guaiacol, N-acetyl-L-tyrosine, and L-cystine.

5,8-Tetradecadienoic acid is the intermediate product of lipid oxidation. Arachidonic acid is an essential fatty acid and it is the bioactive substance of 20-alkyl acid derivatives, including the direct inflammatory precursors of prostaglandin E2 and leukotrienes. These active substances regulate the lipid metabolism and vascular elasticity and activate the platelets. In Addition, these active substances are directly involved in endothelial cells damage, platelet aggregation, thrombus formation, coronary artery spasm, and formation of atherosclerotic plaque, which suggests a close pathological relationship to patients with CHD showing dampness syndrome [16]. Tyramine and guaiacol are metabolic products of tyrosine, which is closely related to the synthesis of catecholamine.

Hence, according to the etiology and pathogenesis theory of TCM, CHD and CRF share common syndromes that damp-turbidity could lead to the incidence of diseases. From the view of clinical symptoms, patients usually have greasy fur on tongue and heavy sensation in the limbs and body, with nausea, vomiting, and other complaints and signs. However, using metabolomics approaches, differential metabolites were found from dampness syndrome of CHD and CRF, which indicated that dampness syndromes in different diseases were different.

There were 4 common metabolites, that is, phosphate acid, L-phenylalanine, acetoacetic acid, and cystathionine ketimine, found in the serums of patients with CHD showing dampness syndrome and patients with CRF showing dampness syndrome.

Phosphate acid is one of most common metabolites in organism and involved in lots of metabolic pathways. Phenylalanine is one of the essential amino acids. Acetoacetic acid is the intermediate product of fatty acid oxidative decomposition. Accumulation of cystathionine ketimine can aggravate the myocardial damage. These metabolites illustrate that there are common metabolic disorders of amino acids and lipid in patients with dampness syndrome along with CHD and CRF. Additionally, these metabolites could be the fundamental molecules that cause dampness syndrome in two diseases. In order to validate the pathological role of these molecules in dampness syndrome of various diseases precisely, more researches are needed to be carried out in future.

## 6. Conclusion

Our results demonstrated that the metabolomics method is one of the effective tools to study “same syndromes in different diseases.” Currently, quantitative analysis with these metabolites has not yet been conducted. Therefore, larger amount of samples would be collected to examine the contents of metabolites quantitatively and explore the possibility of using these metabolites as biomarkers to assist disease early diagnosis and help TCM syndrome differentiation and therapeutic valuation. The omics techniques provide a powerful tool to reveal the nature of “same syndromes in different diseases” for dampness syndrome, and it also provides theoretical foundations for personalized and precise therapy of TCM.

## Figures and Tables

**Table 1:** Differential peaks existing in the serums of patients with CHD showing dampness syndrome compared to the serums of healthy subjects.

*M*/*Z*	RT	VIP	*P*
130.0630	672.6	2.09887	1.02*E* − 12
224.0797	586.8	2.01341	1.01*E* − 09
143.0582	684.0	1.99692	4.44*E* − 16
318.2559	588.0	1.98661	1.22*E* − 14
226.0590	618.0	1.97223	1.09*E* − 10
281.1124	751.2	1.94525	2.31*E* − 12
160.0372	2172.0	1.66067	3.25*E* − 10
106.0773	1800.0	1.61730	4.21*E* − 09
192.0270	843.0	1.58200	8.18*E* − 12
89.9045	506.4	1.50676	3.98*E* − 07

**Table 2:** Differential peaks existing in the serums of patients with CRF showing dampness syndrome compared to the serums of healthy subjects.

*M*/*Z*	RT	VIP	*P*
130.0630	672.0	1.78563	1.11*E* − 15
169.9988	699.0	1.69143	2.66*E* − 15
267.0968	688.2	1.68281	3.99*E* − 15
143.0582	684.0	1.64337	1.00*E* − 15
126.0429	853.8	1.64025	1.24*E* − 10
167.0582	699.6	1.63299	1.50*E* − 10
142.0378	937.8	1.60282	4.44*E* − 16
181.0739	929.4	1.57410	8.88*E* − 16
156.0171	708.0	1.57345	8.13*E* − 11

**Table 3:** Differential peaks existing in the serums of patients with CHD showing dampness syndrome compared to the serums of patients with CRF showing dampness syndrome.

*M*/*Z*	RT	VIP	*P*
300.2089	844.2	2.11428	4.13*E* − 02
137.0841	507.0	1.79156	2.37*E* − 02
224.1776	864.0	1.78254	2.69*E* − 02
192.0270	841.8	1.66977	1.20*E* − 04
299.0866	876.0	1.71287	3.67*E* − 02
304.2402	2070.0	1.63517	2.20*E* − 04
124.0524	505.2	1.59766	5.50*E* − 04
223.0845	705.0	1.47564	2.55*E* − 02
240.0217	714.0	1.46718	4.22*E* − 02

**Table 4:** Differential metabolites existing in the serums of patients with CHD showing dampness syndrome compared to the serums of healthy subjects.

*M*/*Z*	Formula	Metabolites
130.0630	C_6_H_10_O_3_	2-oxo-3-Methylvaleric acid
224.0797	C_10_H_12_N_2_O_4_	3-Hydroxy-DL-kynurenine acid
143.0582	C_6_H_9_NO_3_	Vinylacetylglycine
318.2559	C_21_H_34_O_2_	Pregnenolone
226.0590	C_9_H_10_N_2_O_5_	Nitrotyrosine
281.1124	C_11_H_15_N_5_O_4_	1-Methyladenosine
160.0372	C_6_H_8_O_5_	Oxoadipic acid
106.0773	C_3_H_6_O_4_	Glyceric acid
192.0270	C_6_H_8_O_7_	Citric acid
89.9045	C_4_H_8_O_2_	Isobutyric acid

**Table 5:** Differential metabolites existing in the serums of patients with CRF showing dampness syndrome compared to the serums of healthy subjects.

*M*/*Z*	Formula	Metabolites
130.0630	C_6_H_10_O_3_	2-oxo-3-Methylvaleric acid
169.9988	C_3_H_7_O_6_P	Dihydroxyacetone phosphate
267.0968	C_10_H_13_N_5_O_4_	Adenosine
143.0582	C_6_H_9_NO_3_	Vinylacetylglycine
126.0429	C_5_H_6_N_2_O_2_	Imidazoleacetic acid
167.0582	C_8_H_9_NO_3_	Pyridoxal
142.0378	C_5_H_6_N_2_O_3_	5-Hydroxymethyluracil
181.0739	C_9_H_11_NO_3_	L-Tyrosine
156.0171	C_5_H_4_N_2_O_4_	Orotic acid

**Table 6:** Differential metabolites existing in the serums of patients with CHD showing dampness syndrome compared to the serums of patients with CRF showing dampness syndrome.

*M*/*Z*	Formula	Metabolites
300.2089	C_20_H_28_O_2_	Retinoic acid
137.0841	C_8_H_11_NO	Tyramine
224.1716	C_14_H_24_O_2_	5,8-Tetradecadienoic acid
192.0270	C_6_H_8_O_7_	Citric acid
299.0866	C_10_H_13_N_5_O_6_	8-Hydroxyguanosine
304.2402	C_20_H_32_O_2_	Arachidonic acid
124.0524	C_7_H_8_O_2_	Guaiacol
223.0845	C_11_H_13_NO_4_	N-Acetyl-L-tyrosine
240.0217	C_6_H_12_N_2_O_4_S_2_	L-Cystine

**Table 7:** Metabolic pathways involved in parts of differential metabolites from serums of patients with CHD and CRF showing dampness syndrome.

Metabolite	Pathway
Tyramine	Tyramine metabolism
Arachidonic acid	Arachidonic acid metabolism

**Table 8:** Same metabolites existing in the serums of patients with CHD showing dampness syndrome and the serums of patients with CRF showing dampness syndrome.

*M*/*Z*	RT	VIP	Formula	Metabolites
97.9769	753.0	0.218203	H_3_PO_4_	Phosphoric acid
165.0790	521.4	0.287885	C_9_H_11_NO_2_	L-Phenylalanine
102.0317	876.0	0.309992	C_4_H_6_O_3_	Acetoacetic acid
203.0252	1789.8	0.312836	C_7_H_9_NO_4_S	Cystathionine ketimine

**Table 9:** Metabolic pathways involved in parts of the same metabolites from serums of patients with CHD and CRF showing dampness syndrome.

Metabolite	Pathway
L-Phenylalanine	Phenylalanine and tyrosine metabolism

## Abbreviations

CHD: Coronary heart disease
CRF: Chronic renal failure
TCM: Traditional Chinese Medicine
NMR: Nuclear magnetic resonance
GC-MS: Gas chromatography and mass spectrometry
LC-MS: Liquid chromatography and mass spectrometry
PLS-DA: Partial least square discriminant analysis
TCA: Tricarboxylic acid cycle.

## References

[1] Hood L., Heath J. R., Phelps M. E., Lin B. (2004). Systems biology and new technologies enable predictive and preventative medicine. *Science*.

[2] Nicholson J. K., Wilson I. D. (2003). Understanding ‘global’ systems biology: metabonomics and the continuum of metabolism. *Nature Reviews Drug Discovery*.

[3] Nicholson J. K., Holmes E., Lindon J. C., Wilson I. D. (2004). The challenges of modeling mammalian biocomplexity. *Nature Biotechnology*.

[4] Mao J., Niu Z., Boli Z. (2011). Literature analysis of studies on the TCM syndromes of coronary heart disease in the recent 40 years. *Journal of Traditional Chinese Medicine*.

[5] Zhong J., He L., Ding X. (2006). Study on clinical syndrome classification and related biochemical indexes in 146 cases of chronic renal failure. *Journal of Traditional Chinese Medicine*.

[6] Joint Group of Clinical Names of the International Society of Cardiology (1979). Nomenclature and diagnostic criteria of ischemic heart disease. *Circulation*.

[7] Professional Group Kidney Disease (1993). Summary of the symposium on classification and diagnostic criteria of primary glomerular diseases. *Chinese Journal of Internal Medicine*.

[8] National Kidney Foundation (2002). K/DOQI clinical practice guidelines for chronic kidney disease: evaluation, classification and stratification. *American Journal of Kidney Diseases*.

[9] Chinese society of integrated traditional and Western Medicine (1991). Coronary heart disease in TCM of chest pain or chest tightness, heart palpitations, shortness of breath and fatigue syndrome standard. *Chinese Journal of integrated traditional and Western Medicine*.

[10] Zheng X. (2002). *Chinese Traditional Medicine New Drug Clinical Research Guiding Principle*.

[11] He L., Cheng Y., Liao P. (2010). Exploring the prediction model of chronic renal failure based on serum proteomics. *Basic & Clinical Medicine*.

[12] Qian P., Bai G., Zhao J., Wang Y. (2012). Metabonomics studies on urine specimens from patients with chronic renal failure. *Chinese archives of Traditional Chinese Medicine*.

[13] Yao D., Wang Y., He L. (2016). Serum proteomics analysis for patients with acute myocardial infarction with syndrome of phlegm and blood stasis. *Chinese Journal of Traditional Chinese Medicine and Pharmacy*.

[14] He L., Cheng Y., Liao P. (2010). Exporing on differential diagnosis model of damp-syndrome of TCM in chronic renal failure based on serum proteomics. *Chinese Journal of Traditional Chinese Medicine and Pharmacy*.

[15] Pu Z., Liping M., Hu J. (2010). Study on relationship between age, hs-CRP level and sex hormone in Postmenopausal women with coronary artery disease. *hinese Journal of Geriatric Heart Brain and Vessel Diseases*.

[16] Liu H., Li R. (2005). Prostaglandins and coronary heart disease. *Chinese Journal of Gerontology*.

